# Family happiness and college students’ smartphone addiction control: the chain mediation effect of emotion regulation and self-control

**DOI:** 10.3389/fpubh.2024.1444033

**Published:** 2024-10-09

**Authors:** Xiangju Yin, Yongli Yu, Hongwei Qian, Zixu Wang

**Affiliations:** ^1^School of Emergency Management, Henan Polytechnic University, Jiaozuo, China; ^2^Safety and Emergency Management Research Center, Henan Polytechnic University, Jiaozuo, China

**Keywords:** family happiness, self-control, emotional regulation, smartphone addiction, chain mediation effect

## Abstract

**Background:**

More and more college students use smartphones for a long time every day. The problem of smartphone addiction has become a hot topic of social concern. It not only affects the physical and mental health of college students but also affects the development of families and society. To investigate the effect of family happiness on college students’ smartphone addiction, we conducted a questionnaire survey and analysis.

**Methods:**

In this study, 214 college students were investigated using the adolescent family happiness questionnaire, college students’ smartphone addiction scale, self-control scale, and emotion regulation scale. The data were analyzed by Amos and SPSS26.0 software.

**Results:**

The results showed that: (1) Family happiness, emotion regulation, and self-control were significantly inversely correlated with smartphone addiction; (2) Emotion regulation and self-control served as partial mediators in the linkage between family happiness and smartphone addiction, and the chain mediation effect of emotion regulation and self-control was significant.

**Conclusion:**

Family happiness, emotion regulation, and self-control are key factors that significantly influence college students’ smartphone addiction behavior. Family happiness not only has a direct effect on college students’ smartphone addiction behavior but also has an indirect effect on it through the chain mediation effect of emotion regulation and self-control.

## 1 Introduction

By the end of 2023, there are 1.092 billion Internet users in China, and the Internet penetration rate has reached 77.5% (1). As a mobile terminal of the Internet network, smartphones are becoming increasingly important in people’s daily life because of their convenient use and diverse functions. More and more people are over-relying on smartphones and using them irrationally. Xu et al. (2) found that 93.1% of college students were *dependent on smartphones*, and only 6.9% were *non-dependent*. Smartphone addiction has become a hot topic of social concern, which not only impinges negatively on the physical and mental health of individuals but also has an influence on many aspects such as family, society and the economy. Hence, it is imperative to delve into the underlying causes and mechanisms of smartphone addiction.

The status of the family is very important. It is not only a warm harbor for us, but also can inspire everyone to pursue self-improvement and obtain more happiness. Family happiness can provide good psychological support for college students, thereby enhancing their happy mood. Adolescents who have chronically experienced a deficiency in family happiness are prone to smartphone addiction as a means of detaching from familial misfortune and emotional stress, thereby mitigating the negative emotions and experiences stemming from the distress and tension encountered within their family and emotional environments (3). Wang et al. (4) conducted a questionnaire survey to show that the parents’ education methods for their children affected the development of children’s personality characteristics and interpersonal relationships. College students were cultivated under inappropriate family education models are prone to various adaptive disorders of smartphone addiction when faced with a more complex social environment. On the contrary, good communication between parents and college students, positive parent–child relationships, and supportive parental supervision can greatly help college students use smartphones wisely (5). It appears that family happiness may be a pivotal factor in the control of smartphone addiction among college students. This study attempts to explore the impact of family happiness on smartphone addiction among college students, and *what path* does *family happiness* affect college students’ smartphone addiction behavior, to provide a scientific basis for family education, school management and social intervention in college students’ smartphone addiction, and promote the healthy growth of college students and social development.

## 2 Literature review and research hypothesis

### 2.1 Definition of concepts

Smartphone addiction is also known as *smartphone dependence*, *problematic smartphone use*, *excessive use of smartphone* etc., it mainly shows that the individual loses control over the use of the smartphone. Experts have proposed numerous definitions to encapsulate the concept of smartphone addiction. Liu et al. (6) and Zhong et al. (7) characterized smartphone addiction as a type of behavioral dependency, wherein smartphones offer users a suite of personalized products that closely align with their psychological needs, affording them a heightened sense of enjoyment and immersion that is derived from their use of the smartphone. Smartphone features will inevitably promote the use of smartphones and even addiction. Based on behavioral characteristics, Lee et al. (8) suggested that smartphone addiction has the characteristics of tolerance, withdrawal, prominence, loss of control, craving, and excessive use of smartphones that interfere with an individual’s daily life. According to the consequences of the impact, Lepp et al. (9) found that smartphone use can have a negative impact on face-to-face communication, leading to a decrease in the quality of communication, a decrease in intimacy, and even conflict. At the same time, some studies have also pointed out that smartphone dependence is highly correlated with the frequency of social media use, which may lead users to focus too much on virtual social interactions and ignore real social interactions, thus affecting the development of interpersonal relationships. In short, the problem of smartphone addiction has attracted more and more attention. We need to deeply understand the negative impact of smartphone addiction on us in order to formulate corresponding strategies to reduce the occurrence of smartphone addiction.

### 2.2 Family happiness and smartphone addiction

**Figure 1:**
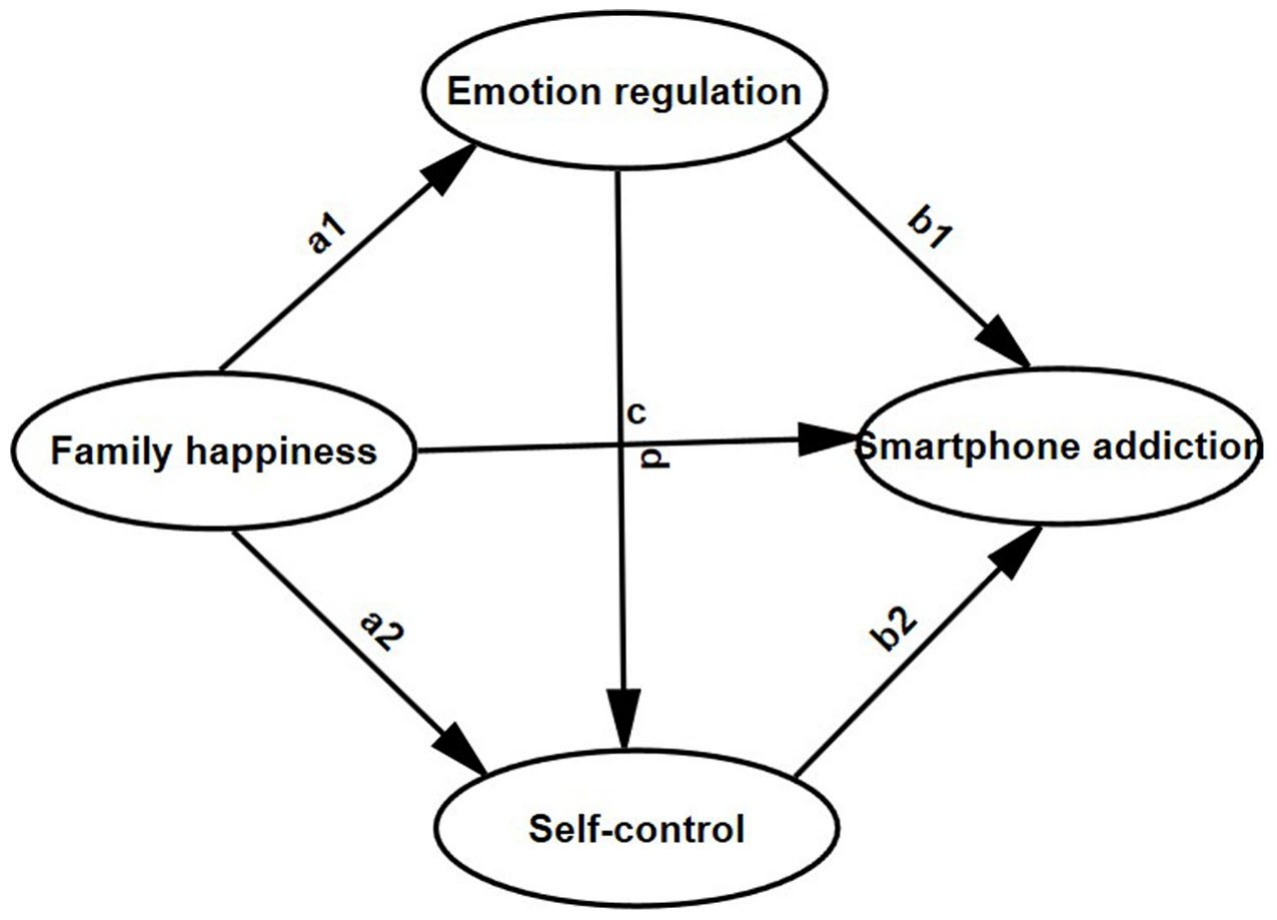
A hypothesis chain mediation model of family happiness and smartphone addiction.

Family happiness is closely related to the overall functioning of the family system. The family systems theory posits that the family is conceptualized as a system of interplay and interconnection. Thus, the dynamics and interactions among family members exert a significant influence on the behaviors and developmental trajectories of individuals (10). If individuals encounter severe conflicts within the family, a fragile family structure, and strained parent–child relationships, these elements may diminish their perception of family happiness. Factors influencing family happiness can have an impact on the propensity for smartphone addiction among college students (11, 12). Therefore, it is possible to enhance the interaction mode between family members, the degree of communication and support within the family and other factors to improve family happiness, decrease college students’ reliance on smartphones, and lower the incidence of addiction. Accordingly, the hypothesis of this study, H1 family happiness is proposed as a possible inverse predictor of smartphone addiction (as shown in Figure 1, path c).

### 2.3 Smartphone addiction and other factors

Smartphone addiction is influenced not only by objective circumstances but also by individual subjective factors. Self-control remains a focal point of scholarly inquiry. According to Baumeister et al. (13), Self-control is when an individual becomes aware that their actions will impact their goals, and he can alter and regulate such behavior through certain means. Luo et al. (14) define self-control as the process by which individuals consciously overcome impulses, habits, or automatic reactions and adjust their behavior to achieve long-term goals. The level of self-control may be an important factor influencing an individual’s smartphone addictive behavior (15). Self-control theory holds that self-control is a finite resource that people may deplete when faced with temptations and impulses, leading to a weakening of self-control. In the face of smartphones, if individuals lack self-control, they are prone to fall into overuse behaviors, resulting in a tendency to become addicted to smartphones (16). However, there is little research on whether family happiness can enhance people’s self-control ability so that they can self-regulate and suppress impulses and reduce the likelihood of addiction when faced with the temptation of smartphones.

In addition, studies have found that deficits in emotion regulation are associated with problematic social media use. Emotional regulation refers to a range of ways for people to manage their emotions when they feel changes in their emotions to keep themselves mentally healthy. Gross, a well-known psychologist, proposed that people’s emotional control may be affected by a variety of factors (17). The most important of these is that people control their emotions by improving their behavior, feelings, and thinking. In addition, people may also improve their emotional state by improving their motivation and suppressing energy. Individuals who suffer from negative emotions such as anxiety and depression in the family for a long time have difficulty in emotional regulation. The college students facing stressful life events are more inclined to adopt non-adaptive strategies. Non-adaptive strategies are significantly positively correlated with problematic smartphone addiction. Although people’s emotional regulation and emotional health are directly affected by the family environment (18, 19), further investigation is warranted to determine whether enhancing family happiness can subsequently bolster emotional regulation and, in turn, impact smartphone addiction.

In summary, an individual’s smartphone addiction is influenced by a variety of factors. Studies have indicated a close relationship between family happiness, emotional regulation, self-control, and adolescent smartphone addiction. Nevertheless, the researches tend to focus on the impact of a single factor, with limited consideration given to the influence of combined factors on college students’ smartphone addiction behaviors. Based on the above analysis, we take family happiness as an objective factor and emotion regulation and self-control as subjective factors to explore how they affect the smartphone addiction behavior of college students. Specifically, this study explores how the objective factor, *family happiness,* and subjective factors, *emotion regulation and self-control,* affect the smartphone addiction behavior of college students, aiming to gain a more profound understanding of the underlying causes and development process of college students’ smartphone addiction. Consequently, the hypotheses are posited as follows: H2: Family happiness may serve as a negative predictor of smartphone addiction via emotional regulation (Figure 1, paths a1 and b1); H3: Family happiness may function as a negative predictor of smartphone addiction through self-control (Figure 1, paths a2 and b2); and H4: Family happiness may act as a negative predictor of smartphone addiction by way of emotional regulation and self-control (Figure 1, paths a1, d, and b2).

## 3 Materials and methods

### 3.1 Subjects of survey

The survey was administered from March 1, 2024, through March 30, 2024, within Henan Province, China. To minimize sampling error, a random sampling method was employed to select five universities (in a ratio of 11:1) from a total of 60 universities in Henan Province. Each university distributed between 40 and 50 questionnaires based on the student population and grade distribution to ensure participant diversity. Investigators were selected from the five universities and underwent standardized training. These investigators disseminated the digital questionnaires to the participants via a provided link. Respondents accessed the questionnaire through the Questionnaire Star platform, completed their responses, and upon submission. The questionnaires were automatically compiled for collection. A total of 220 questionnaires were gathered, of which 214 were deemed valid, resulting in an efficacy rate of 97.27%. In this research, the independent variable was family happiness, the dependent variable was smartphone addiction, and the mediating variables included emotional regulation and self-control. Using G-power software, the effect size was set to 0.15, the statistical strength value was set to 0.95, and a significance level value was set to 0.05. It was calculated that the sample size required for this survey was at least 119 people. The experimental sample in this study met the sample size calculated by G-power software. Inclusion Criteria: (1) The participants have read the informed consent form and voluntarily participate in the study; (2) The participants have basic cognitive ability and can understand the meaning of each item in the questionnaire; (3) The respondents completed the online questionnaire by himself or with the help of the investigators. Exclusion Criteria: (1) non-native speaking participants; (2) The questionnaire was incomplete or has logical errors. The questionnaire was administered in Chinese, mainly including sociodemographic characteristics, family characteristics, time allocated to smartphone use, adolescent family happiness scale, college students’ smartphone addiction scale, emotional regulation scale, self-control scale, and other contents. The participants in this survey comprised 111 males (51.9%) and 103 females (48.1%), with an average age of 22 years (SD = 2.51).

### 3.2 Research tools

#### 3.2.1 Adolescent family happiness scale

This investigation utilized the adolescent family happiness scale developed by Zhang (20), comprising 29 items across five dimensions: satisfaction with family member relationships, satisfaction with family educational approaches, satisfaction with familial communication, positive emotional experiences within the family, and negative emotional experiences within the family. Rated on a five-point scale, where 1 denotes complete dissatisfaction and 5 denotes complete satisfaction, higher scores reflect a higher index of family happiness among participants. Internal consistency reliability of the scale in this measurement was 0.96 and Cronbach’s *α* coefficient was 0.92.

#### 3.2.2 College students’ smartphone addiction scale

The college students’ smartphone addiction scale was developed by Su (21). This scale comprises 22 items, covering six dimensions: withdrawal behavior, salience behavior, social comfort, negative effects, use of App, renewal of App. Rated on a five-point scale, where 1 indicates very inconsistent and 5 indicates very consistent, the scale measures the extent of smartphone addiction among college students. Through calculation, internal consistency reliability of this scale in this measurement was 0.97, and Cronbach’s *α* coefficient was 0.96.

#### 3.2.3 Self-control scale

Yi (22) modified the self-control scale originally developed by Tangney et al. (23), which comprises 13 items. This scale encompasses both self-regulatory behavior and impulse control, employing a five-point rating system where 1 denotes complete inconsistency and 5 indicates complete consistency. Notably, four of these items were reverse-scored. Consequently, after inverting the scores for these four items, a higher overall score reflects a greater degree of self-control in the participants. Internal consistency reliability of the scale in this measurement was 0.97 and Cronbach’s *α* coefficient was 0.95.

#### 3.2.4 Emotional regulation scale

The research employed a revised version of the emotion regulation scale by Wang (24), initially formulated by Gross et al. (17). From the six subscales within the comprehensive scale, the emotional regulation strategies scale was chosen for this study, encompassing a total of eight items. Each item was rated on a seven-point Likert scale, where 1 signified complete disagreement and 7 indicated complete agreement. The aggregate score for emotion regulation was obtained by summing the scores of all item. Internal consistency reliability of the scale in this measurement was 0.96, and Cronbach’s *α* coefficient was 0.96.

### 3.3 Data processing and common method bias test

The structural equation model was constructed based on Amos. The correlation analysis, descriptive statistics and normality test were carried out on the data by SPSS26.0. For self-reported data, there may be an issue of common methodological bias, and therefore there is a need to assess whether there is common methodological bias between the variables involved in the study. Confirmatory factor analysis was used to test for common method bias for all self-assessment items. Set 1 common latent variable, if the model can fit effectively, it is considered there were common methodological biases, and the results showed that the data and the model fit poorly (*χ^2^/df* = 12.974, NFI = 0.698, RFI = 0.644, IFI = 0.713, CFI = 0.713, RMSEA = 0.237). Therefore, the data collected in this study did not present serious methodological bias issues.

## 4 Results

### 4.1 Description and Pearson correlation analysis

Table 1 presents the mean, standard deviation, correlation analysis, and normality test outcomes for the data. As indicated in Table 1, there are significant pairwise correlations among family happiness, emotion regulation, self-control, and smartphone addiction. A significant negative correlation is observed between family happiness and smartphone addiction. Conversely, there are significant positive correlations between family happiness and both self-control and emotion regulation. Additionally, smartphone addiction is significantly negatively correlated with both self-control and emotion regulation. A significant positive correlation exists between self-control and emotion regulation. Because the Structural Equation Model relies on the maximum likelihood estimation method to estimate the model parameters. The maximum likelihood estimation method is optimal when the data satisfies a normal distribution. Normality testing through skewness and kurtosis is a way to detect whether the data deviates from a normal distribution. Kline (25) considered that if the absolute value of the skewness coefficient remains below 3 and the absolute value of the kurtosis coefficient is confined to less than 8, then the data can be deemed suitable for statistical analysis based on the assumption of normal distribution. The outcomes presented in Table 1 indicate that both the skewness and kurtosis for each dimension fall within permissible limits. This suggests that the data pertaining to the measured variables adhere to an approximately normal distribution. Consequently, the sample data are deemed appropriate for subsequent statistical examination.

**Table 1:** Descriptive statistics, partial correlation analysis, and normality test outcome variables for each study variable.

Variables	M	SD	Family happiness	Smartphone addiction	Self-control	Emotional regulation	Skewness	Kurtosis
Family happiness	3.25	0.82	1	—			2.37	3.80
Smartphone addiction	3.15	0.88	−0.72**	1	—		0.76	3.65
Self-control	2.84	0.92	0.075**	−0.67**	1	—	0.13	3.59
Emotional regulation	3.62	1.21	0.072**	−0.62**	0.66**	1	0.15	3.79

**means *p* < 0 0.01; *means *p* < 0.05.

### 4.2 The association between family happiness and smartphone addiction: a chain mediated effect analysis

To investigate the mediating effects of emotional regulation and self-control on the association between family happiness and smartphone addiction, this research evaluated five models, as outlined in Table 2 (comprising the hypothetical model, the full mediation model, and three partial mediation models). As illustrated in Table 2, the hypothetical model demonstrated superior fit compared to the other four models (*χ^2^/df* = 2.23, NFI = 0.95, RFI = 0.94, IFI = 0.97, CFI = 0.97, RMSEA = 0.07). Consequently, the data model employed in this study exhibits robust validity.

**Table 2:** Fitting index of each model.

Mode	Path	χ^2^/df	NFI	RFI	IFI	CFI	RMSEA
Hypothetical model	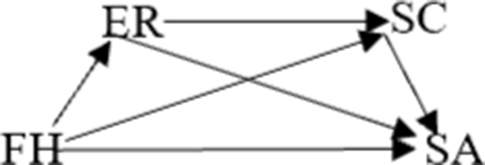	2.23	0.95	0.94	0.97	0.97	0.07
Fully mediated model	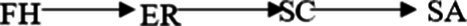	4.51	0.90	0.88	0.92	0.90	0.13
Part of the mediation model 1	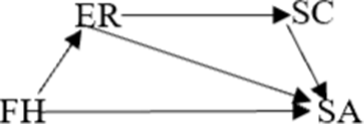	3.82	0.92	0.90	0.94	0.94	0.12
Part of the mediation model 2	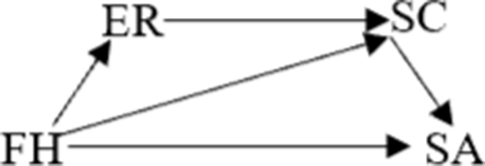	3.72	0.92	0.90	0.94	0.92	0.11
Part of the mediation model 3	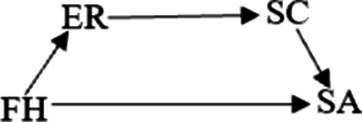	4.20	0.91	0.89	0.93	0.93	0.12

In the path description, FH is Family happiness; ER is Emotional regulation; SC is Self-control and SA is Smartphone addiction.

**Figure 2:**
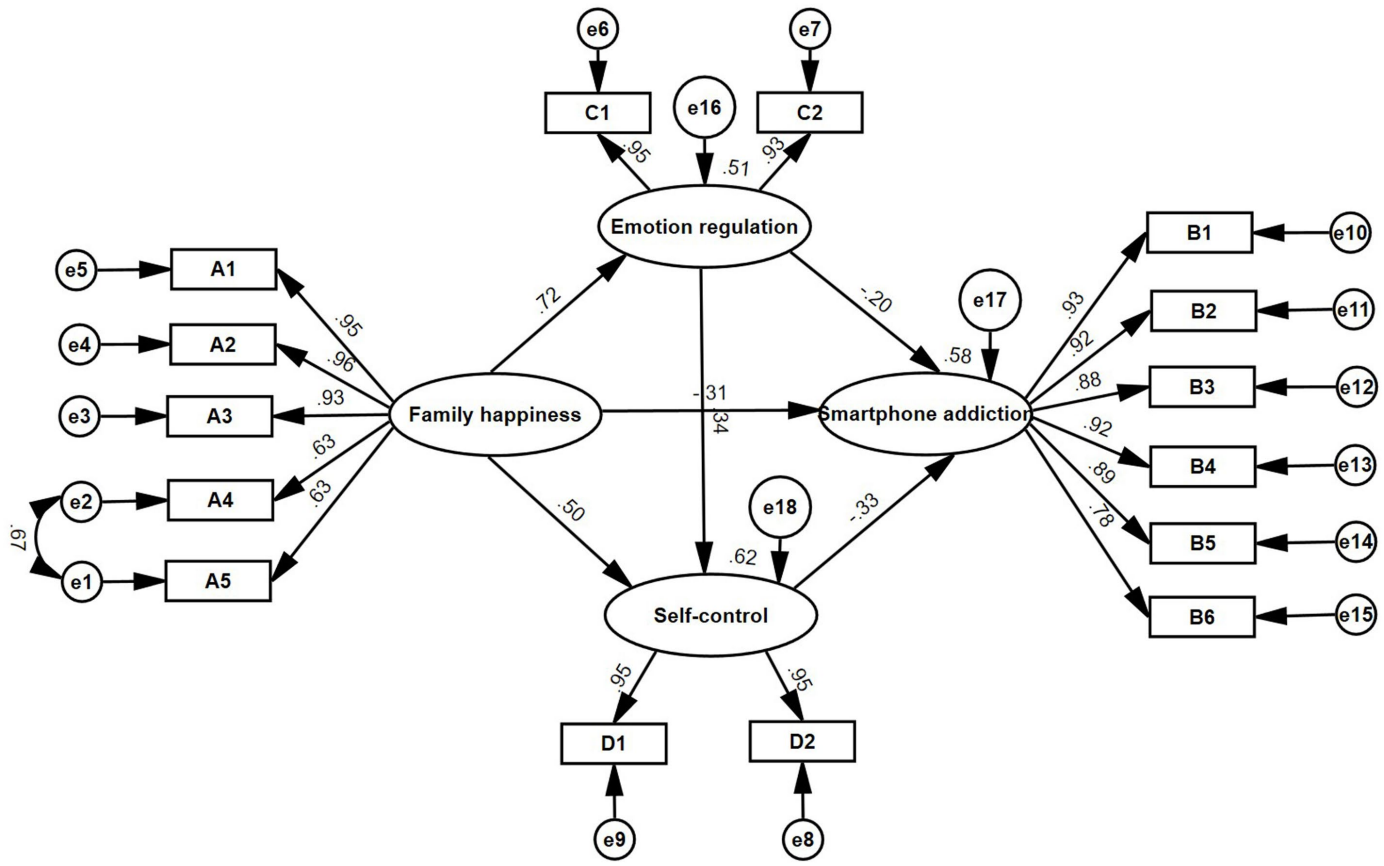
Chain mediation model of family happiness and smartphone addiction between emotion regulation and self-control.

To delve into the relationship between family happiness and smartphone addiction, this study posited *family happiness* as the independent variable, with *self-control* and *emotional regulation* serving as mediating variables, and *smartphone addiction* as the dependent variable, thereby constructing a chain mediation model. In conjunction with the model modification indices from the AMOS output, the model was refined by introducing the covariance path, thereby enhancing the model’s structural validity. The ultimate findings are depicted in Figure 2. The standardized fit indices for each path yielded satisfactory metrics. Notably, family happiness [*β* = −0.31, *t* = −3.58, *p* < 0.001, *CI*: (−0.62, −0.19)] emerged as a significant negative predictor of college students’ smartphone addiction behavior, substantiating hypothesis H1. Emotion regulation [*β* = −0.20, *t* = −2.53, *p* < 0.05, *CI*: (−0.25, −0.03)] and self-control [*β* = −0.33, *t* = −3.93, *p* < 0.001, *CI*: (−0.45, −0.13)] also significantly predicted smartphone addiction.

To quantify the indirect effect value of each path, the custom syntax in Amos software was employed to separately encode all paths, the mediation effect between variables was assigned, and the chain mediation effect was tested based on the bias correction percentile Bootstrap method. The computation was iterated 5,000 times with a 95% confidence interval, and the interval excluded zero. Following execution, the mediated effects of the pathways were obtained. The specific results are shown in Figure 2 and Table 3. The detailed findings are depicted in Figure 2 and Table 3. Family happiness exerts its influence on smartphone addiction via mediators—emotional regulation and self-control—with respective effect sizes of 20.3% and 23.5%. Consequently, Hypotheses H2 and H3 are validated. Additionally, family happiness can also impact the smartphone addiction behaviors of college students through a sequential mediation involving both emotional regulation and self-control, with an effect size comprising 11.4% of the overall effect and confidence intervals ranging from −0.20 to −0.42. Hence, Hypothesis H4 is substantiated.

**Table 3:** Bootstrap analysis of mediated effect.

Effect	Mediation paths	Estimate	Effect size (%)	Confidence interval (95%) [LLCI, ULCI]
Direct effects	Family happiness → Smartphone addiction	−0.39	44.8%	[−0.62, −0.19]
	Family happiness → Emotional regulation → Smartphone addiction	−0.18	20.3%	[−0.32, −0.03]
Mediation effect	Family happiness → Self-control → Smartphone addiction	−0.21	23.5%	[−0.36, −0.09]
	Family happiness → Emotional regulation → Self-control → Smartphone addiction	−0.10	11.4%	[−0.42, −0.20]
Total mediation effect		−0.48	55.2%	[−0.68, −0.30]

## 5 Discussion

We find that family happiness, emotion regulation and self-control are important factors influencing college students’ smartphone addiction behavior. Family happiness can inversely predict college students’ smartphone addiction behavior. Emotion regulation and self-control play a partial mediating role between family happiness and smartphone addiction. The chain mediation effect of emotion regulation and self-control is significant. The results indicate that family happiness not only directly affects the smartphone addiction behavior of college students, but also indirectly regulates smartphone addiction through the chain mediation of emotion regulation and self-control.

### 5.1 Relationship between family happiness and smartphone addiction

The results suggest that family happiness can inversely predict the smartphone addiction behavior of college students. That is, the higher the family happiness index, the less addiction to smartphones. Family is the most important source of moral and material support for individuals. When the family is dysfunctional, it is often difficult for individuals to obtain the social support brought by good family happiness. They may use smartphones that provide online social support as their main channel for seeking happiness. Eventually, it can lead to problematic smartphone usage behavior (26, 27). Smartphone addiction can increase loneliness in individuals, which in turn reduces subjective well-being (28). Family systems theory views the family as an interdependent and mutually influencing whole, and the interaction between family members is important for individual behavior and development. Therefore, it is possible to reduce the occurrence of smartphone addiction among college students by improving the interaction mode between family members, enhancing the degree of communication and support within the family, improving the family atmosphere, improving family happiness, and establishing positive emotional connections.

### 5.2 The mediated role of self-control

This study reveals that family happiness can indirectly affect college students’ dependence on smartphones through self-regulation. Studies have shown that family happiness and self-control are closely related (29, 30). According to the view of self-control theory (31), when an individual encounters a conflict between the two modes of automatic and controlled behavior, the self-control function can help the individual reduce the consumption of resources and achieve the set goal. Part of the reason why family happiness is significantly negatively correlated with smartphone addiction is that family happiness enhances the self-discipline of individuals. Wilhelm et al. (32) suggested that emotional support, information, etc., provided by family members can often be used as an external resource to cope with negative behaviors. In addition, studies have also found that warm, supportive families can provide a sense of belonging, helping individuals improve their levels of self-control (33). When an individual’s control mode of thinking and action is enhanced, it can help him get rid of his out-of-control behavior (34). When the individual’s self-control ability is high, the individual can be prompted to self-plan, self-execute, self-evaluate, self-motivate, self-correct, etc. (35). Through the dynamic change of self-regulation and restraint, the behavior and degree of smartphone addiction of college students can be finally reduced.

### 5.3 The mediated role of emotional regulation

The investigation of the relationship between family happiness and smartphone addiction also found that family happiness can indirectly predict the smartphone addiction behavior of college students through emotional regulation. Emotions directly reflect the individual’s family happiness. Positive emotions are the experience of family happiness. Negativity is a manifestation of family unhappiness (36). When individuals make situational choices, they often draw on the views and opinions of their parents and important others in their surroundings. However, poor parent–child relationships or poor relationships with others, or even emotional dysregulation problems among parents, can make it difficult for individuals to receive timely and accurate feedback when faced with situational choices. Which can lead to these individuals not being able to effectively master the skills of using adaptive emotion regulation strategies, thus trying to reduce negative emotions by using smartphones (37, 38). Previous studies have found that family happiness has a significant negative predictive effect on negative emotions (39). According to developmental psychopathology, the overlap period between early adulthood and college students is a critical period for the shaping of mental health (40). If college students do not experience more family happiness during the critical period. This can lead to a lack of healthy physical and mental development. When faced with negativity, their resources are insufficient to maintain internal balance. They may seek new external pathways to regulate their emotions, leading to negative behaviors such as smartphone addiction.

### 5.4 The mediated effect of emotional regulation and self-control in the relationship between family happiness and smartphone addiction

The study confirms that emotion regulation and self-control not only play a moderating role in the relationship between family happiness and smartphone addiction but also can form a chain mediation role of family happiness → emotion regulation → self-control → smartphone addiction.

First of all, self-control has a negative predictive effect on smartphone dependence. Individuals with strong self-control ability are more able to control impulses and desires. Mobile games, chat apps, short videos, video apps, and shopping apps on smartphones can quickly and long-lastingly attract college students. Individuals who lack self-control are more likely to become addicted to these functions of smartphones. Because they can get immediate satisfaction from using their smartphones. People with strong self-control are often able to control smartphone addiction because they can compare and analyze instant gratification and long-term goals, and choose long-term goals between them. Secondly, the family environment has a direct impact on people’s emotional well-being and emotional regulation. In a harmonious family environment, if individuals feel a stronger sense of security, this can help to enhance family satisfaction and reduce their negative emotions (41). People with high family happiness tend to have positive emotions in their hearts, and this determines their high self-control level (42, 43), thereby reducing smartphone addiction.

## 6 Suggestion

In this study, family happiness is used as the independent variable. Self-control and emotion regulation are used as the mediating variables to construct a chain mediation model. The role of family happiness in college students’ smartphone addiction is clarified. It provides a certain theoretical basis and solution for guiding college students to reduce smartphone addiction.

Improving family happiness is an important way to solve the problem of smartphone addiction among college students. A family with a high level of family happiness can provide good emotional support to family members. In a harmonious family environment, family members can build a close communication bridge. Family members can confide in each other about their worries and joys. Family members caring and understanding each other can reduce college students’ reliance on smartphones for emotional support. In addition, family education is also an important aspect of enhancing value and happiness, through which family members can understand the dangers of smartphone addiction and help family members establish an active and healthy lifestyle.

Weak self-control is an important factor that leads college students to fall into smartphone addiction. Therefore, it is of great significance to strengthen the self-control ability of college students to reduce the degree of smartphone addiction. First of all, families and schools can develop a clear incentive and punishment mechanism to inhibit smartphone addiction. A reasonable system is an effective way to personal behavior guidance, which can enhance the self-control of college students. In addition, cultivating the self-monitoring ability is also an important aspect of strengthening self-control. Family members can set specific rules with college students to reduce smartphone time. Schools can help students distance themselves from smartphones by encouraging them to participate in team projects and activities, fostering their self-supervision and teamwork.

Emotion regulation is the ability of individuals to manage and regulate their emotions, which is of great significance for reducing the degree of smartphone addiction. Good emotion regulation can help people better cope with negative emotions and stress, and reduce their dependence on smartphones as an emotion regulation tool. Therefore, families can enhance college students’ awareness of emotions by encouraging them to communicate with their families and share their emotions. Schools and society can provide training in emotion management so that college students can master the skills to express emotions and enhance emotional regulation. This reduces their smartphone addiction.

## 7 Limitations of the study

This investigation validates the association between family happiness and smartphone addiction, yet it was not without its limitations. First, this survey relies on the online Questionnaire Star platform and the sample size was small. To strengthen the authenticity and accuracy of the data, the sample size will be increased and face-to-face interviews will be conducted in future studies. Second, Due to the cross-sectional investigation, the results revealed the qualitative relationship between the variables, but it was not possible to measure the degree of control of each variable for smartphone addiction. Therefore, there is a further need for additional interventions to assess quantitative change. Third, this study did not consider other potential variables, such as social support, personality traits, academic pressure, etc. Therefore, more influencing factors need to be explored in the following research. It will provide more ways to prevent and reduce the mobile phone addiction of college students.

## Data Availability

The raw data supporting the conclusions of this article will be made available by the authors, without undue reservation.
